# *Streptomyces Levis ABRIINW111* Inhibits SW480 Cells Growth by Apoptosis Induction

**DOI:** 10.15171/apb.2018.076

**Published:** 2018-11-29

**Authors:** Behnaz Faramarzian Azimi Maragheh, Parisa Fatourachi, Seyede Momeneh Mohammadi, Behnaz Valipour, Meysam Behtari, Alireza Dehnad, Hojjatollah Nozad Charoudeh

**Affiliations:** ^1^Stem Cell Research Center, Tabriz University of Medical Sciences, Tabriz, Iran.; ^2^Tabriz Higher Education Institute of Rab-Rashid, Department of Biological Sciences, Tabriz, Iran.

**Keywords:** Anti-Cancer, Colon cancer, Metabolites, Streptomyces, SW480

## Abstract

***Purpose:*** Streptomyces sp., a dominant genus in Actinomycetes, is the source of a wide variety of secondary metabolites. Microbial metabolites can be utilized as novel anticancer agents; with fewer side effects. The present article illustrated the anti-carcinogenic effect of the ether extracted organic metabolites derived from Streptomyces bacteria on SW480 colon cancer cell line.

***Methods:*** MTT assay was performed in order to investigate the cytotoxicity effect of metabolites on SW480 cells. Apoptosis and cell cycle arrests were measured by flowcytometry. Morphological changes were indicated by Propidium iodide staining andP53 gene expression was evaluated by real-time PCR.

***Results:*** Streptomyces Levis ABRIINW111 inhibited cell growth, increased Caspases 3 and reduced Ki67 expression in a concentration/time-dependent manner in SW40 cells. Metabolites increased subG1 phase (apoptosis) and also cell cycle arrest in G1, G­­­2/M and S phase. P53 gene expression followed Sw480 cells treatment significantly.

***Conclusion:*** Streptomyces sp. metabolites have anti-carcinogenic effect on colon cancer cells. Streptomyces Levis ABRIINW111 metabolites are a candidate for Colon cancer treatment.

## Introduction

Colon cancer is one of the most prevalent malignancies in the world.^1,2^ Neoplastic cells reduced normal growth and exhibit abnormal cell cycle. Sporadic and somatic genes mutations are mostly involved in colon cancer.^3,4^ It was indicated that natural products are able to induce apoptosis in cancer cells without serious side effects.^5^ Over the decades, considerable efforts were put into the use of natural products as the anticancer agents against various types of cancers in order to activate cell death signals in cancer cells.^6^ Previous studies have shown that natural products have therapeutic effects such as anti–‌cancer and anti-infection activity.^7^ Actinomycetes are the best source of bioactive secondary metabolites for utilizing in drug discovery and biotechnology.^8^*Streptomyces* is a dominant genus in Actinomycetes, which is the‌ source of 80% of the produced bioactive secondary metabolites.^9^ For example, Rapamycin- isolated from the soil bacteria; *Streptomyces hygroscopicus*- is an anticancer agent which induces apoptosis and cell cycle arrest. Metabolites of *Streptomyces sp. SY-103* have strong cytotoxic effects which induce apoptosis in human leukemia cells through the activation of caspase3 and inactivation of *Akt* signaling.^10,11^ Recently, it was indicated that ether extracted metabolites of *Streptomyces Levis ABRIINW111* have anti-carcinogenic effects on colon cancer^12^ as well. Apoptosis was used as a target for cancer therapy and several drugs were designed to activate Caspase family.^13^ Caspases family are the key elements in apoptosis and are influenced by both intrinsic and extrinsic pathways.^14^*P53* is one of the most important genes in apoptosis which has a critical role in cell cycle.^15^ It can cause cell cycle arrest in certain stages of cell cycle by up regulation of both P21 and P27 protein that consequently inhibits all cyclin–CDK complexes and can result in apoptosis.^16^

In the present study, a new strain of *Streptomyces* - isolated from the Zagros Mountains Hamadan in Iran is reported. The mentioned strain produced secondary metabolites against gram positive and gram negative bacteria.^17^ Based on GeneBank data-base-, there is 98% similarity between 16S rDNA gene and *S. Levis* strain NRRL B-16370. Bergey’s manual of systematic bacteriology strongly suggested that morphology properties of strain *ABRIINW111* belonged to the genus Streptomyces.^17^ The extracted metabolites had anti-cancer effect on Colon cancer by cell growth inhibition, arresting cell cycle, inducing apoptosis and by increasing *P53* expression in Colon cancer. 

## Materials and Methods

### Microbial culture and Fermentation

*Streptomyces Levis ABRIINW111* strain -isolated from soil samples- was extracted in the Department of Microbial Biotechnology, AREEO, Tabriz, Iran and was cultured in Nutrient Agar medium (70148, Sigma, Germany) at 29 °C for 7 days. The loops full of bacteria were inoculated into 25 ml of Mueller Hinton Broth medium (70192, Sigma, Germany) and incubated while agitating on shaker incubator set as 70 rpm at 29 °Cfor 36 h.^17^ After fermentation time, 1 ml of pre-culture was applied to inoculate 1,000-ml Erlenmeyer flasks, each containing 150 ml of fresh Mueller Hinton Broth medium. The fermentation was done at 29 °C for 7 days on shaker incubator set as 70 rpm, centrifuged at 4000 rpm for 20 min. The Cell free filtrate was mixed with equal volume of Diethyl ether (1:1 V/V) shaken for 10 min at 175 rpm, extracted by Diethyl ether (100921, Merck, USA), by the use of separating funnel. Finally, the obtained organic extract was undertaken to be concentrated at room temperature to achieve 0.01 gr crude extract which was maintained at 4 °Cuntil being utilized.^17^ As previously described, the turbidity 620 nm, 0.08 O.D. was considered appropriate for inoculation.^17^

### Metabolites analysis with HPLC method

**Figure 1:**
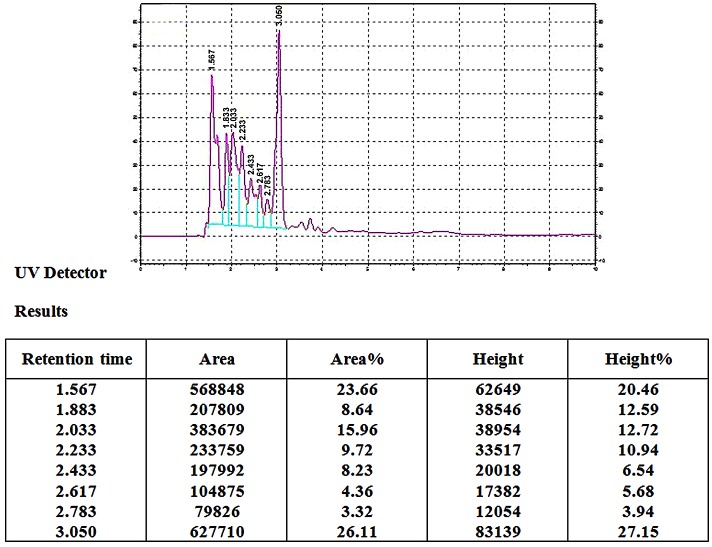
HPLC Chromatogram of Diethyl ether extracted metabolites of *Streptomyces Levis ABRIINW111*

Extracted metabolites were analyzed by HPLC method. The column system consisted of a C18 column, UV detector and 215 nm wave lengths. Dried metabolites were dissolved in acetonitrile. The mobile phase consisted of methanol, H_2_o and acetonitrile (45, 50, 5). Injected metabolites were 1 µl and the flow rate was set as 1 ml min^-1^ for 10 min. Peak responses were measured at 215 nm.^17^(Figure 1)

### Cell culture and MTT assay

Colon cancer cell line, SW480, was purchased from Iran National Cell Bank (Pasteur Institute/IRAN). The cells were cultured in RPMI-1640 medium (51800-035, Gibco, USA) supplemented with 10% Fetal Bovine Serum (10270-106, Gibco, USA), 100 U ml^-1^ Penicillin and 100 μg ml^-1^ streptomycin (15140-122, Gibco, USA).1×10^4^ SW40 cells were seeded per well in 96-well plates with 100μl of culture medium and were incubated for 24 h. Metabolites were diluted in culture medium with less than 0/1% DMSO (1029521000, Merck, USA) and the cells were incubated with various concentration of metabolites (100, 500, 1000, 2000 and 5000 ng ml^-1^) for 24, 48 and 72 h. The highest concentration of DMSO was employed as positive control. After incubation time, supernatant was carefully replaced with 20 µL of MTT reagent (M6494, Sigma, Germany) {3-(4,5-dimethyl thiazol-2-yl)-2,5-diphenyl tetrazolium bromide ( 5 mg ml^-1^), incubated at 37 °C for 4 h and 100 µL of DMSO was subsequently added to dissolve the emerged colored formazan crystals. The optical density was measured at 570 nm with a reference wavelength of 630 nm by micro plate Elisa reader (Biotek ELx 808, USA). The effect of metabolites was calculated as a percentage of treated cells/control cells. Cytotoxicity rate was expressed as IC50 value (the concentration caused 50% reduction in cell survival compared to the control cultured in parallel without drug) calculated by Microsoft Excel (2010).

#### Fluorescent-based staining

In order to confirm the cell death, the cells morphology changes were analyzed by the Acridine Orange/Ethidium Bromide double staining method. A number of 10^4^ SW480 cells were seeded into each well of chamber slide (30118, SPL, Korea) and incubated for 24 h in a humidified, 5% CO_2_ at 37 °C. The cells were treated with various concentration of metabolites, stained with 10 μl Acridine Orange 50 mg ml^-1^ (A6014, Sigma, Germany) and Ethidium Bromide 50 mg ml^-1^ (E7637, Sigma, Germany) for 5 min and were washed with PBS. Ultimately, the cell death was characterized under a fluorescence microscope with 400× magnification.

#### Flow cytometry analysis for cell cycle and apoptosis

SW480 cells were treated with metabolites (100, 500, 1000 ng ml^-1^) for 48 h, the harvested cells were washed with ice-cold PBS, fixed in 70% (v/v) ethanol (100983, Merck, USA) and stored at 4°C for 1 h. Then the cells were transferred into PBS, incubated with 5μl (50 mg ml^-1^) RNase A for 30 min at 37C, stained with 50 µg ml^-1^ Propidium Iodide**(11348639001,** Sigma, Germany) for 30 min. Furthermore, the cell cycle profile was determined by BD FACS Calibur system and analyzed with FlowJo software. For evaluating apoptosis, harvested cells were permeabilized with Cytofix/Cytoperm(51-6896KC, BD Bioscience, USA) solution in 4 °C for 20 min, washed with Perm/wash(51-6897KC BD Bioscience / USA) solution and incubated with monoclonal antibodies including Monoclonal Anti-Caspase 3(51-68655X, eBioscience, USA) and Monoclonal Anti- Ki-67 (12-5699-42, eBioscience, USA) for 20 min. Consequently, the cells were washed with PBS and finally analyzed by BD FACSCalibur employing FlowJo software.

#### Real time PCR

SW480 cells were treated with 1000 ng ml^-1^ of metabolites for 48 h, Total RNA were extracted by using RNX plus kit ( RN7713C, Sina Clon, IRAN) and 1μg of total RNA was transcribed reversely into single stranded cDNA using Bioneer cDNA syntheses kit (K-2261-6, Bioneer, Korea) according to manufacturer's instruction. RT-PCR was performed by utilizing the SYBR Green real-time PCR kit (7571540RXN, BD Bioscience, USA) in 14 μl reaction volume, which contained 7 μl of SYBR Green Master Mix PCR, 0/6 p mole form each forward and reverse primers (F:5^'^-GTTCCGAGAGCTGAATGAGG-3' and R: 5'- TTATGGCGGGAGGTAGACTG-3'), 1 μl of diluted cDNA template and 5/4 μl of DEPC treated water. The conditions for amplification of genes were in order: initial denaturation at 95 °C for 3 min, 40 cycles of denaturation at 95 °C for 15 s, annealing at 60 °C for 60 s and elongation at 72 °C for 5 min. A quantitative real-time PCR was performed with Rotor-Gene 6000 and the relative gene expression level was determined by employing Rotor-Gene 6000 series software 1/7.

## Results and Discussion

### Streptomyces Levis ABRIINW111 induced apoptosis and reduced proliferation in SW480

Actinomycetes and their dominant genus* Streptomyces sp.* play an important roles as an anti-cancer agent.^18,19^ Recent studies reported anti-tumor activity of the *Streptomyces sp. SY-103* metabolites by inducing apoptosis in Leukemia and Hela cells.^20^

**Figure 2:**
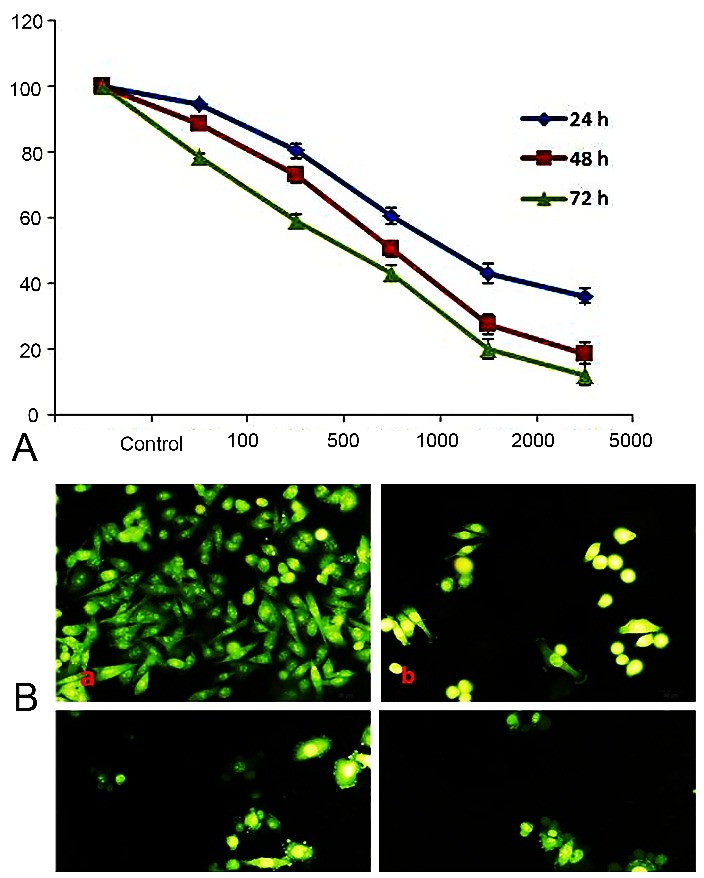
Cell cytotoxicity was examined by MTT assay. (a) SW480 cells were incubated with the indicated concentrations of *Streptomyces Levis* metabolites for 24, 48 and 72 h (absorbance at a wave length of 570-630 nm). (b) Morphology of SW480 cells after 48 h exposure to Metabolites of *Streptomyces sp. ABRIINW111* was examined by fluorescent scope. Untreated cells (a) treated cells 100 ng/ml (b) treated cells 500 ng/ml (c) treated cells 1000 ng/ml (d). After 48 h, treated cells were revealed as the marked blebbing morphology. Values are expressed relatively to the control and as Mean ± SD of three independent experiments (P < 0.001).

The cytotoxicity effect of metabolites on SW480 cell line was determined by MTT assay. The viability of SW480 cells was decreased after treatment with 100, 500, 1000, 2000 and 5000 ng ml^-1^ of metabolites in 24, 48 and 72 h. As indicated in (Figure 2a), metabolites inhibited the cells growth rate in a concentration and time-dependent manner. The cells viability in concentration of 5000 ng ml^-1^ for 24, 48 and 72 h treatment was 36/25%, 18/75% and 12/25%, respectively. The IC_50_ value in 24, 48 and 72 h treatment was approximately 1100, 1000, 900 ng ml^-1^. Therefore, the cell viability was decreased significantly by the increasing of time and metabolites concentration (Figure 2a).

Half of the discovered bioactive secondary metabolites are derived from Actinomycets^18^ which able to act as antibiotics and anti-tumor agents.^9,18,21,22^

Moreover, it was proved that *Streptomyces Levis ABRIINW111* inhibited cell growth in a concentration/time-dependent manner and the mentioned metabolite has anti-tumor activity against colon cancer.

Cancer cells were incubated with metabolites in concentration of 100,500,1000 ng ml^-1^ for 48 h and double staining was performed by Acridine Orange/Ethidium Bromide to analyze the morphological transformations by florescent microscopy (Figure.2b). Apoptotic changes were measured in concentration of 100, 500 and 1000 ng ml^-1^ by plasma membrane blebbing. The concentration of 1000 ng ml^-1^, increased the plasma membrane blebbing in SW480 cells‌ significantly (Figure 2b).

**Figure 3:**
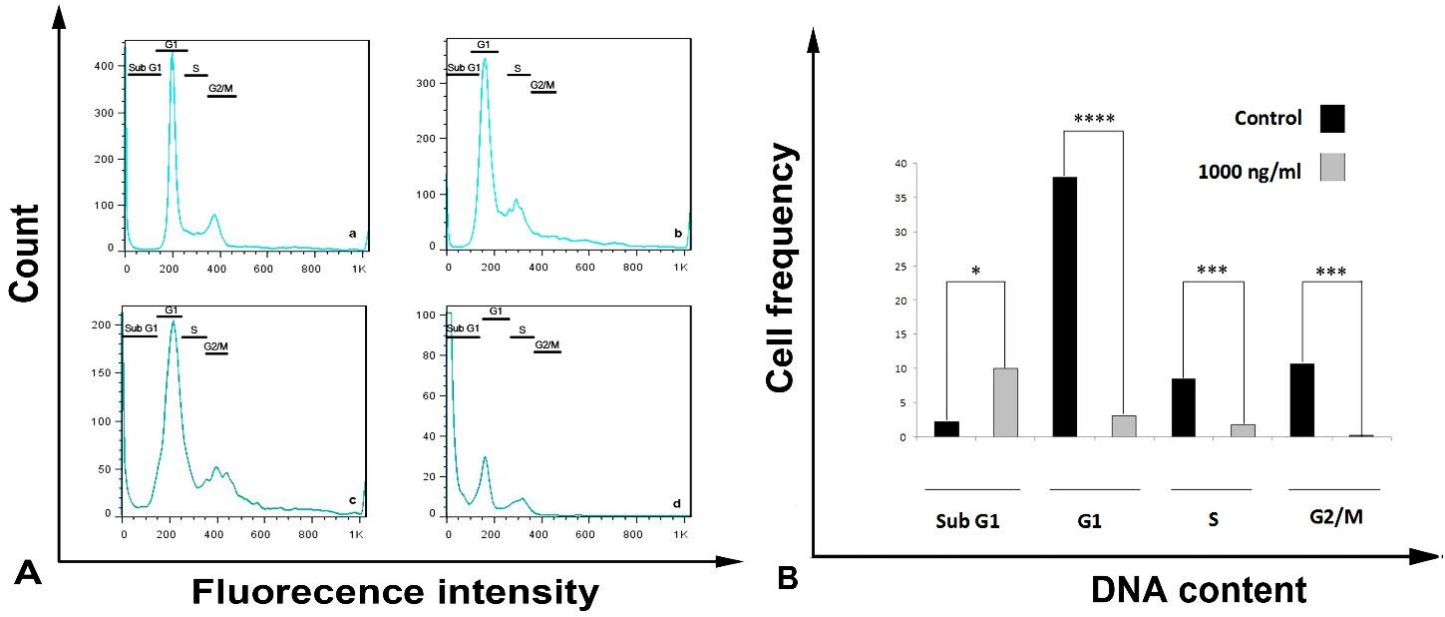
The cell cycle analysis following SW480 cell treatment with metabolites for 48 h. (a) Flow cytometry data: Untreated cells (control) (a) 100 ng ml^-1^ (b) 500 ng ml^-1^ (c) 1000 ng ml^-1^ (d). (b) Mean (SD) proportion of cell cycle. Mean± SD from 3 independent experiments (Pvalue <0.0001)

The cell cycle was analyzed in sub G1, G1 and G2/M phases (Figure 3).* Streptomyces Levis ABRIINW111* metabolites in concentration of 1000 ng ml^-1^increased G1 and G2/M phase whereas an average of S phase was reduced. However, subG1 phase increased from 2/6% in control to 11/8 % in treated cells. G_1_ phase reduced dramatically from 38/9% to 3/42 % in treated cells and also G_2_/M phase was 0/29% in treated cells and 10/9% in the control group‌ (Figure 3).

Therefore cell cycle analysis indicated that metabolites are able to increase subG1 phase (apoptosis) and cell cycle arrest in G_1_, G_2_/M and S phase as well.

Apoptosis is an active process that leads to cell death and is mediated by two extracellular and intracellular signaling pathways, where Caspases family is involved in both of them.^16,23^

**Figure 4:**
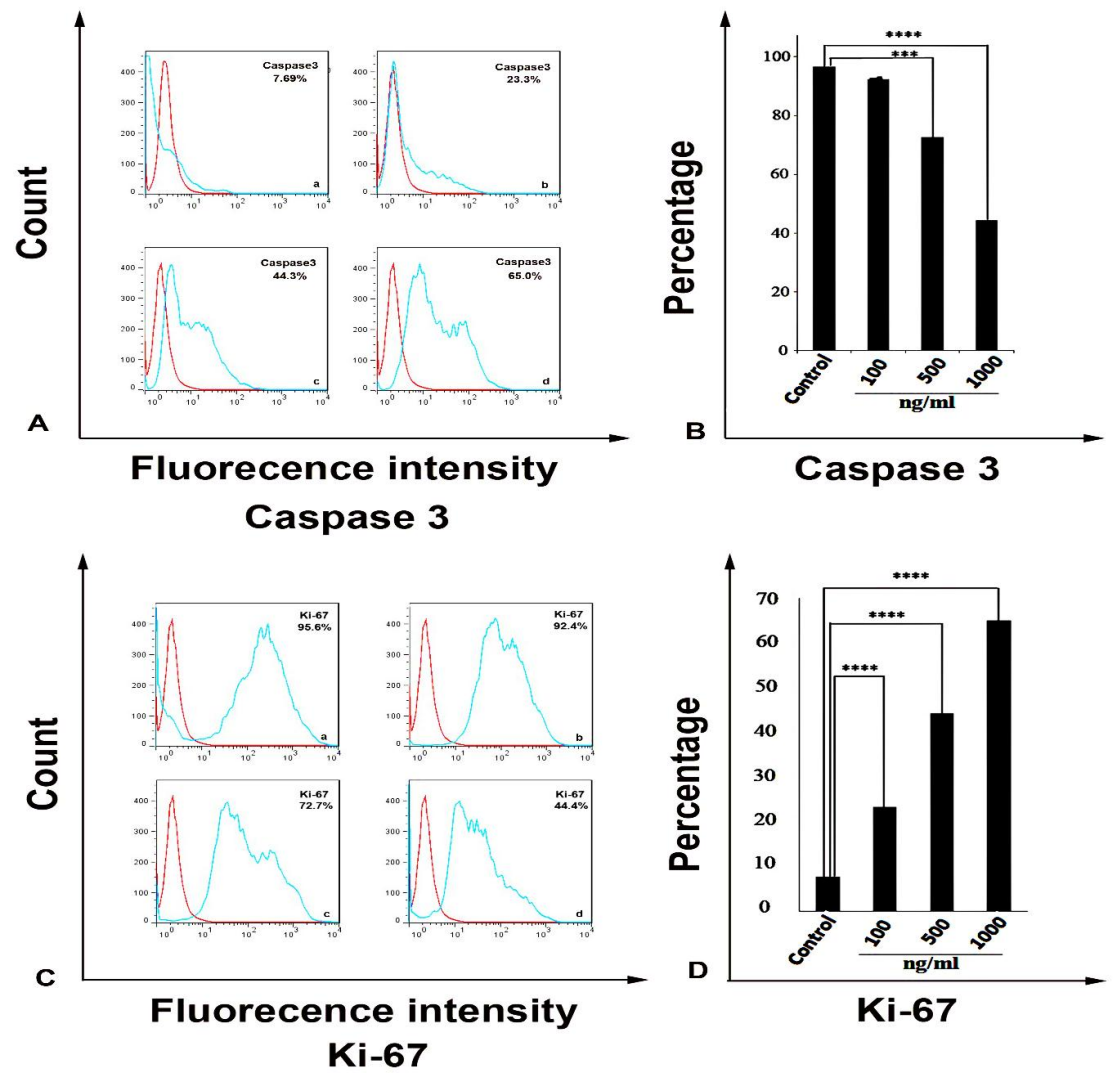
*Streptomyces Levis ABRIINW111* can alter the apoptosis and proliferation of colon cancer cells. SW480 was treated with indicated concentrations of metabolites for 48 h and was evaluated by flow cytometry (FACS) for Caspase3 and Ki-67 expression. (a) FACS profile of Caspase 3 expression: Control cells (a) 100 ng ml^-1^ (b) 500 ng ml^-1^ (c) 1000 ng ml^-1^ (d).(b) Mean (SD) proportion of Caspase 3 expression with an indicated concentration of metabolites. (c) FACS profile of Ki67 expression: Control cells (a) 100 ng ml^-1^ (b) 500 ng ml^-1^ (c) 1000 ng ml^-1^ (d). (d) Mean (SD) proportion of Ki67 expression with the indicated concentrations of metabolites. Values are demonstrated as mean ± SD from 4 independent experiments (P_Value <0.0001).

In order to express the effects of metabolites on proliferation and apoptosis, proliferation (Ki-67 expression) and apoptosis (Caspase 3 expression) were evaluated by flow‌cytometry. In accordance with morphological changes, metabolites increased Caspase3 expression and reduced Ki67 expression in concentration dependent manner. After 48 h, Caspase3 level was‌ 23/3%,‌‌ 44/3%,‌‌ 65/0% in concentration of 100, 500 and 1000 ng ml^-1^respectively, whereas in non-treated cells it was 7/69% and Ki-67 level in concentration of 100, 500 and 1000 ng ml^-1^was 92/4% ,72/7% and 44/4% respectively, but it was 95/6% in the control group (Figure 4).

Ki-67 expression is low in G_1_ phase and is increased during S and G_2_ phase and reached the highest expression in M phase.^24-26^ Recent studies revealed that Ki-67 percentage reduced after chemotherapy, tamoxifen therapy and chemoendocrine therapy.^27-33^ In cancer cells, Oncogene p53 is frequently mutated or overexpressed. Cancer cells presenting mutation or overexpression of oncogene *P53* are an indication of higher rates of proliferation as measured by Ki-67.^33-37^

### Streptomyces Levis ABRIINW111 metabolites increased P53 gene expression in SW480 cells

**Figure 5:**
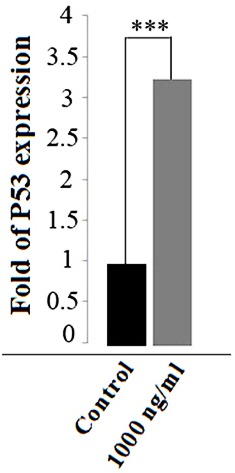
P53 gene expression increased in SW480 cell line using *Streptomyces levis ABRIINW111*. SW480 cells treated with metabolites for 48 h and P53 folds expression measured by real time PCR*.* Value is illustrated as mean ± SD from 3 independent experiment (P value <0.0001).

Previous studies demonstrated that *P53* has a relationship with both apoptosis and cell cycle.^15^ In order to examine *P53* gene expression, a quantitative real-time PCR which followed the incubation of SW480 with 1000 ng ml^-1^ *Streptomyces Levis* metabolites for 48 h was performed. *P53* expression increased remarkably in treated cells around 3-fold in comparison with control cells (Figure 5).

*P53* as a tumor suppressor gene is mutated in a wide variety of human malignancies. It encodes a transcription factor that regulates cellular responses to DNA damage, cell cycle progression, and genomic stability. *P53*-dependent cell cycle arrest can act through the Cdk inhibitor on *P21*. Based on several studies, following DNA damage, *P53* and *P21* mediate G1 and G2 phase of cell cycle will arrest. It was acknowledged that p21 transcription is induced in p53-infected cells.^38-42^

Metabolites might affect phosphorylation/ dephosphorylation of *Cdc2* and may lead to cell cycle arrest in G_2_/M phase.^43-45^ Metabolites inhibit topoisomerase II and can arrest the cell cycle in G_2_/M phase.^46^ In the present study, the subG1 phase was increased, whereas G_1_ phase and G2/M phase were decreased in the treated cells. These results indicated that metabolites could cause a delay in cell cycle at G2/M phase.

The induction of apoptosis with *P53* is a major factor as tumor suppressor.^47^ Metabolites inducing apoptosis may be mediated by Bcl-2 family which either inhibit or activate apoptosis^48^ Bax and Bad as pro-apoptotic effector genes stimulate realizing cytochrome C from the mitochondria which in turn will induce the activation of Caspases resulting in cell death.^49,50^

## Conclusion

The presented study demonstrated that *Streptomyces Levis ABRIINW111* metabolites increased P53 gene expression and apoptosis significantly and reduced the proliferation in SW480 Colon cancer cell line as well. Additionally, the cell cycle was arrested in G_1_ and G_2_/M phase. Overall, *Streptomyces Levis ABRIINW111* metabolites are a candidate for Colon cancer treatment.

## Acknowledgments

Authors gratefully acknowledge Kobra Velaei, Soheila Montazersaheb, Mehdi rasouli *and* Dariush Shanehbandi for the technical support. The current study was supported by Research Council of Tabriz University of Medical Sciences (grant 5/104/1236).

## Ethical Issues

The current article does not contain any studies with human or animal subjects.

## Conflict of Interest

All authors declared that there is no conflict of interest.
